# The vulnerability and jubilation of publication; a reflection on publishing with MedEdPublish

**DOI:** 10.15694/mep.2020.000029.1

**Published:** 2020-02-13

**Authors:** Lorna Davin

**Affiliations:** 1 University of Notre Dame

**Keywords:** Publication, vulnerability, reviewers, feedback, constructive, robust, transparent, community of practice

## Abstract

This article was migrated. The article was marked as recommended.

Promoting your research findings to the broader academic and clinical community requires publication dependent on peer review.

This opinion piece is a short reflection on the author’s journey as she completes her PhD and traverses the fickle world of peer review and publication.

Feeling deflated after a poor journal review, the author briefly explores the vulnerability of rejection, and the rewarding consequences of publishing on-line with MedEdPublish, as an open access journal, where post-publication reviews are transparent and there is an opportunity to engage in a shared dialogue with members of the community of practice.

## The vulnerability and jubilation of publication; a reflection on publishing with MedEdPublish

When completing a PhD you really are undertaking an apprenticeship in research; it is a beginning not an end in itself. You hope to contribute an original contribution to the field, in my case, as a qualitative researcher, to build understanding and in the long run, improve the doctor’s journey in the hope of ultimately improving the patient experience. Promoting your research outcome to the broader academic and clinical community requires publication dependent on peer review.

Rigorous supervision, demanding milestones and the scrutiny of internationally renowned examiners prepares you little for the publication journey. After submitting to a recognized journal and receiving a less than favourable review of my 5000 word summary of my PhD - where I agonized over what to and what not to include, of my 80,000 word thesis - I felt a total failure, a fraud. It was with enormous trepidation I picked myself up, brushed myself off and carried on ... determined that the interns’ voices in my study be heard.

Publishing with MedEdPublish, also has its fair share of vulnerability. In submitting your article you submit yourself to open, post-publication review from a cadre of reviewers, they comment and rate you online for all to see. There is a potential vulnerability for the reviewer too, as their review may place them at odds with their fellow reviewers.

I remember pressing the final submit button with enormous angst, going to bed with a knot in my stomach, and my eyes squeezed tight with tension, making a promise to myself that I would not look at the results overnight. Tossing and turning, I broke my promise by about 2am!!! To my delight the publication resonated with reviewers and readers. Approximately three hundred reads over the first 24 hours, and constructive feedback, with predominantly positive reviews. The paper, ‘ *Compassion, the first emotion ditched when I’m busy*’. *The struggle to maintain our common humanity,* has gone on to be nudging 5000 reads and 8 reviews, rating an average of 4/5, but more importantly, medical students and interns have shown enormous interest in the journey of the eight interns who I followed over a year. Some have written to me sharing how reading the article has supported them in feeling less alone in their struggle.

Yes, I made the mistake of not providing enough detail of my theoretical approach, in the summarized paper, in preference to sharing the interns’ narrative, and yes, with a few more years’ experience, I understand how I could have better positioned my theoretical approach, but this does not diminish the lived experience of the junior doctors in sharing their journey in their own words.

To receive MedEdPublish’s Best Publication for 2018-19 at AMEE in Vienna, was the surprising icing on the cake, giving me the opportunity to attend AMEE in Glasgow in 2020. I have just returned from the Asia Pacific Med Ed Conference in Singapore after the article won the University of Notre Dame’s National Deputy Vice Chancellor of Academia’s $2000 award for article which ‘most influenced learners’.

So for me, while the feedback from the original journal highlighted where I could improve, MedEdPublish engaged me in a constructive on-line learning dialogue, while providing me with a platform to share my study and the interns’ stories, all within the broad embrace of a supportive, robust, transparent and nurturing community of practice, for which I am very grateful.

## Take Home Messages

• The publication journey is a vulnerable one, where we can learn from the failure of rejection, both individually and collectively
• Learn from your rejections and feedback, but do not allow the process to define your worth as a scholar, or the validity of your findings
• Open access, post-publication peer review, as a progressive alternative model, provides an opportunity for a transparent, shared dialogue, where engagement is encouaged, and learning is nurtured, within a supportive community of practice

## Notes On Contributors

Dr. Lorna Davin is a Senior Lecturer in Medical Education, in the School of Medicine, at the University of Notre Dame Australia (UNDA), Fremantle, Western Australia. She is Program Coordinator for the UNDA Health Professional Education postgraduate suite of courses and teaches into the program. She has over 20 years’ experience in hospital based health professional education. She also has community and local government experience working in aged care, disability and sexuality. Lorna completed her PhD in medical education with the University of Queensland, School of Medicine. She is a qualitative researcher who uses narrative inquiry. Her area of interest is in affective learning, the hidden curriculum, compassion and self-compassion in healthcare - for both patients and their doctors. She is a regular presenter at state and national conferences, has contributed several publications to the field and supervised a range of students undertaking research in medical and health professional education.

ORCID iD: https://orcid.org/0000-0002-4833-4208

## Declarations

The author has declared that there are no conflicts of interest.

## Ethics Statement

Not required as this is an opinion piece.

## External Funding

This article has not had any External Funding

