# Supplementary material for: The vulnerability and jubilation of publication; a reflection on publishing with MedEdPublish
Source: MedEdPublish (2016). 2020 Feb 13;9:29. [Version 1] doi: 10.15694/mep.2020.000029.1 (PMC10697522; doi:10.15694/mep.2020.000029.1)
Supplement: Peer review history

# Reviewer report: Reviewer response for version 1

**DOI:** 10.21956/mep.19998.r30460

**Published:** 2020-02-27

**Authors:** P Ravi Shankar

**Affiliations:** 1 American International Medical University

This review has been migrated. The reviewer awarded 4 stars out of 5 This is an interesting read. The author has described the vulnerability and jubilation of publication very well. The challenges of condensing a thesis into a publication has been well brought out. As a researcher who has published and reviewed often I could identify with the author. She has also commented on MedEdPublish and the system of post-publication peer review which it follows. Rating a paper is a challenging task and we may not be able to put ourselves in the position of the researcher and the challenges he or she may have faced to carry out the work. I am of the opinion that MedEdPublish publishes more practical papers and offers a wider range of submissions than more traditional print or online journals. This short article will be of interest to a wide variety of readers.

Reviewer Expertise:

NA

No decision status is available

# Reviewer report: Reviewer response for version 1

**DOI:** 10.21956/mep.19998.r30461

**Published:** 2020-02-21

**Authors:** Trudie Roberts

**Affiliations:** 1 Leeds Institute of Medical Education

This review has been migrated. The reviewer awarded 4 stars out of 5 I enjoyed reading this piece on the emotional investment when submitting your work for publication. I read this piece after looking at an article in Nature entitled 'What to do when you grant is reject" and there are commonalities. https://www.nature.com/articles/d41586-020-00455-0?utm_source=Nature+Briefing&utm_campaign=84628a6daf-briefing-dy-20200218&utm_medium=email&utm_term=0_c9dfd39373-84628a6daf-42542755Perhaps Laura and others could write a follow up on what to do when you get a paper rejected - rejection recovery - as I think this could be very helpful for the medical education community.

Reviewer Expertise:

NA

No decision status is available

# Reviewer report: Reviewer response for version 1

**DOI:** 10.21956/mep.19998.r30462

**Published:** 2020-02-19

**Authors:** Ken Masters

**Affiliations:** 1 Sultan Qaboos University

This review has been migrated. The reviewer awarded 5 stars out of 5 An interesting and useful reflection piece dealing with the ups and downs (but mostly ups) of publishing in MedEdPublish.In this short piece, the author succinctly points to the experience of moving from PhD to publication. The attempt at trying to reduce an 80,000-word PhD to 5,000 words would be too daunting for most (I certainly would never be able to do it), so it is not entirely surprising that the author found it difficult to do, and then had a less-than positive response to her first submission. Most of us have been there with our first and even second and third submissions :-(. The author is to be commended on her resilience.The submission to MedEdPublish had a different response. As an early reader and reviewer of that paper, I was immediately impressed by it, and my overall thought was “People need to read this” – it appears that many of the MedEd community thought much the same, as the number of reads, accolades and responses from other readers can testify.This reflection piece also notes the difficulties and value of the post-publication, open peer-review model followed by MedEdPublish. Traditional education institutions are currently struggling with recognition of such publications, “indexing” system are only now beginning to address it, and reviewers and authors experience the same trepidation indicated by the author. The author’s experience, however, shows that, although the path ahead may be difficult, it is a worthwhile challenge. As both a medical educator and editor of this journal, I am indeed pleased at the authors’ learning experience, which has come as a well-earned reward for courage and perseverance.

Reviewer Expertise:

NA

No decision status is available
